# 1942. Potential Public Health Impact of Bivalent Respiratory Syncytial Virus Prefusion F (RSVpreF) Maternal Vaccine for Prevention of RSV among US Infants

**DOI:** 10.1093/ofid/ofad500.096

**Published:** 2023-11-27

**Authors:** Ahuva Hanau, Kimberly M Shea, Derek Weycker, Mark Atwood, Erin Quinn, Emily Kutrieb, Jessica E Atwell, Alejandro D Cane, Bradford D Gessner, Sarah J Pugh, Amy W Law

**Affiliations:** Policy Analysis Inc., Brookline, Massachusetts; Pfizer Inc., Newton, Massachusetts; Policy Analysis Inc., Brookline, Massachusetts; Policy Analysis Inc (PAI), Boston, Massachusetts; Policy Analysis Inc., Brookline, Massachusetts; Policy Analysis Inc., Brookline, Massachusetts; Pfizer, Flagstaff, Arizona; Pfizer, Flagstaff, Arizona; Pfizer Biopharma Group, Collegeville, Pennsylvania; Pfizer, Inc., Collegeville, Pennsylvania; Pfizer, Inc., Collegeville, Pennsylvania

## Abstract

**Background:**

Respiratory syncytial virus (RSV) is a leading cause of lower respiratory tract illness (LRTI) among young children. A bivalent stabilized prefusion F subunit vaccine (RSVpreF) for pregnant people to protect their infants against RSV-LRTI is currently under review by the US Food and Drug Administration. We evaluated the potential public health impact, measured as the reduction in clinical outcomes and economic costs, of maternal vaccination with RSVpreF for the prevention of RSV-LRTI among US infants.

**Methods:**

A cohort model was employed to depict clinical outcomes and economic costs of RSV-LRTI from birth to age 1 year, lifetime consequences of premature death, and impact of maternal vaccination with RSVpreF among infants. Clinical outcomes were projected (monthly) based on infant age, gestational age in weeks (wGA) at birth, RSV disease and fatality rates, and mother’s vaccination status, and included cases of medically attended RSV-LRTI and RSV-LRTI deaths. Vaccine effectiveness was derived from interim trial analyses and was assumed to vary by clinical presentation (hospital vs. ambulatory), timing of vaccine administration relative to birth, and wGA at birth. Economic costs were generated based on cases and corresponding unit costs. The public health impact of RSVpreF was evaluated assuming year-round use and 100% uptake.

**Results:**

Without use of maternal RSVpreF vaccine, 48,246 hospitalizations, 144,495 emergency department (ED) encounters, and 399,313 outpatient clinic (OC) visits are projected to occur annually among the US birth cohort of 3.7M infants aged < 12 months. Maternal vaccination with RSVpreF resulted in a reduction of 24,520 hospitalizations, 45,957 ED encounters, and 128,745 OC visits, corresponding with a decrease in direct medical costs equal to $691.8 million and indirect (non-medical) costs equal to $110.0 million (Table).

**Table.**
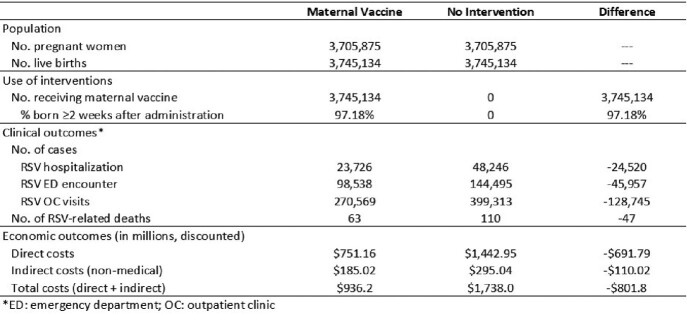
Clinical outcomes and economic costs among US infants aged <1 year with maternal use of RSVpreF vaccine versus no intervention

**Conclusion:**

Results from this evaluation indicate that maternal vaccination with RSVpreF would substantially reduce the clinical and economic burden of RSV-LRTI in infants. These results may be conservative as we did not include potential for reductions in outcomes such as maternal RSV disease, household transmission, and long-term sequelae such as asthma.

**Disclosures:**

**Ahuva Hanau, BS**, Pfizer Inc.: Grant/Research Support **Kimberly M. Shea, Ph.D., M.P.H.**, Pfizer: Employment|Pfizer: Stocks/Bonds **Derek Weycker, Ph.D.**, Pfizer Inc.: Grant/Research Support **Mark Atwood, MS**, Pfizer Inc.: Grant/Research Support **Erin Quinn, BS**, Eisai Inc.: Advisor/Consultant|GlaxoSmithKline: Advisor/Consultant|GRAIL: Advisor/Consultant|Pfizer Inc.: Advisor/Consultant|Santen Pharmaceutical: Advisor/Consultant **Emily Kutrieb, B.A.**, Pfizer Inc.: Advisor/Consultant **Jessica E. Atwell, PhD, MPH**, Pfizer: Stocks/Bonds **Alejandro D. Cane, MD, PhD**, Pfizer: Stocks/Bonds **Bradford D. Gessner, M.D, M.P.H.**, Pfizer: I am an employee of Pfizer|Pfizer: Stocks/Bonds **Sarah J. Pugh, PhD, MPH**, Pfizer Inc: Salary|Pfizer Inc: Stocks/Bonds **Amy W. Law, PharmD**, Pfizer: Employment|Pfizer: Stocks/Bonds

